# State Cannabis Legalization and Cannabis Use Disorder in the US Veterans Health Administration, 2005 to 2019

**DOI:** 10.1001/jamapsychiatry.2023.0019

**Published:** 2023-03-01

**Authors:** Deborah S. Hasin, Melanie M. Wall, C. Jean Choi, Daniel M. Alschuler, Carol Malte, Mark Olfson, Katherine M. Keyes, Jaimie L. Gradus, Magdalena Cerdá, Charles C. Maynard, Salomeh Keyhani, Silvia S. Martins, David S. Fink, Ofir Livne, Zachary Mannes, Scott Sherman, Andrew J. Saxon

**Affiliations:** 1 Columbia University and New York State Psychiatric Institute, New York; 2 Mental Health Data Science, New York State Psychiatric Institute, New York; 3 Health Services Research & Development (HSR&D) Seattle Center of Innovation for Veteran-Centered and Value-Driven Care, Veterans Affairs (VA) Puget Sound Health Care System, Seattle, Washington; 4 Center of Excellence in Substance Addiction Treatment and Education, VA Puget Sound Health Care System, Seattle, Washington; 5 Columbia University, New York, New York; 6 Boston University, Boston, Massachusetts; 7 New York University, New York; 8 VA Puget Sound Health Care System and University of Washington, Seattle, Washington; 9 San Francisco VA Health System and University of California at San Francisco, San Francisco; 10 New York State Psychiatric Institute, New York; 11 VA Manhattan Harbor Healthcare and New York University, New York

## Abstract

**Importance:**

Cannabis use disorder (CUD) is increasing among US adults. Few national studies have addressed the role of medical cannabis laws (MCLs) and recreational cannabis laws (RCLs) in these increases, particularly in patient populations with high rates of CUD risk factors.

**Objective:**

To quantify the role of MCL and RCL enactment in the increases in diagnosed CUD prevalence among Veterans Health Administration (VHA) patients from 2005 to 2019.

**Design, Setting, and Participants:**

Staggered-adoption difference-in-difference analyses were used to estimate the role of MCL and RCL in the increases in prevalence of CUD diagnoses, fitting a linear binomial regression model with fixed effects for state, categorical year, time-varying cannabis law status, state-level sociodemographic covariates, and patient age group, sex, and race and ethnicity. Patients aged 18 to 75 years with 1 or more VHA primary care, emergency department, or mental health visit and no hospice/palliative care within a given calendar year were included. Time-varying yearly state control covariates were state/year rates from American Community Survey data: percentage male, Black, Hispanic, White, 18 years or older, unemployed, income below poverty threshold, and yearly median household income. Analysis took place between February to December 2022.

**Main Outcomes and Measures:**

As preplanned, *International Classification of Diseases, Clinical Modification, *ninth and tenth revisions, CUD diagnoses from electronic health records were analyzed.

**Results:**

The number of individuals analyzed ranged from 3 234 382 in 2005 to 4 579 994 in 2019. Patients were largely male (94.1% in 2005 and 89.0% in 2019) and White (75.0% in 2005 and 66.6% in 2019), with a mean (SD) age of 57.0 [14.4] years. From 2005 to 2019, adjusted CUD prevalences increased from 1.38% to 2.25% in states with no cannabis laws (no CLs), 1.38% to 2.54% in MCL-only enacting states, and 1.39% to 2.56% in RCL-enacting states. Difference-in-difference results indicated that MCL-only enactment was associated with a 0.05% (0.05-0.06) absolute increase in CUD prevalence, ie, that 4.7% of the total increase in CUD prevalence in MCL-only enacting states could be attributed to MCLs, while RCL enactment was associated with a 1.12% (95% CI, 0.10-0.13) absolute increase in CUD prevalence, ie, that 9.8% of the total increase in CUD prevalence in RCL-enacting states could be attributed to RCLs. The role of RCL in the increases in CUD prevalence was greatest in patients aged 65 to 75 years, with an absolute increase of 0.15% (95% CI, 0.13-0.17) in CUD prevalence associated with RCLs, ie, 18.6% of the total increase in CUD prevalence in that age group.

**Conclusions and Relevance:**

In this study of VHA patients, MCL and RCL enactment played a significant role in the overall increases in CUD prevalence, particularly in older patients. However, consistent with general population studies, effect sizes were relatively small, suggesting that cumulatively, laws affected cannabis attitudes diffusely across the country or that other factors played a larger role in the overall increases in adult CUD. Results underscore the need to screen for cannabis use and CUD and to treat CUD when it is present.

## Key Points

**Question:**

What was the role of medical and recreational cannabis law enactment in the nationally increasing rates of cannabis use disorder (CUD) from 2005 to 2019 in Veterans Health Administration patients?

**Findings:**

In this observational study of sequential yearly Veterans Health Administration electronic health record data from 2005 to 2019, CUD rates increased from 1.38% to 2.25% in states with no cannabis legalization, 1.38% to 2.54% in states that legalized medical use, and 1.39% to 2.56% in states that legalized recreational use. Significant but small effect sizes were found for medical and recreational legalization, accounting for 4.7% and 9.8% of the overall increases in the respective states.

**Meaning:**

Although legalization contributed to increasing CUD rates, the role of the laws in these increases may not have been not state-specific or other factors may have played a larger role.

## Teaser

This observational study assesses the role of medical cannabis law and recreational cannabis law enactment in diagnosed cannabis use disorder prevalence among Veterans Health Administration patients by using yearly electronic medical record data from 2005 to 2019.

## Introduction

Cannabis is a widely used psychoactive substance.^1^ While many individuals can use cannabis without harm, 20% to 33%^2^ of individuals who use cannabis develop cannabis use disorder (CUD),^3^ which is characterized by problematic use, clinically significant distress or impairment, symptoms including tolerance, withdrawal, and neglect of other activities,^4^ and psychosocial and health-related problems.^5,6,7,8^

Adult CUD rates have increased in the US general population,^9,10^ inpatients,^11^ and veterans,^12,13^ amidst declining perceptions of cannabis risk,^14,15^ increasing cannabis potency,^16^ and legalization. As of November 17, 2022, 37 US states had enacted medical cannabis laws (MCLs) and 21 states and Washington, DC, had enacted recreational cannabis laws (RCLs). MCLs and RCLs could increase rates of CUD by decreasing perceptions of cannabis risk and increasing availability and commercialization.^1,17,18^ MCL and RCL have played a significant role in the increased prevalence of adult cannabis use,^19,20,21^ but few studies examined the role of MCL or RCL in the national increases in adult CUD rates. In analyses of 3 US surveys conducted between 1991 and 2013, the prevalence of adult CUD increased more after MCL enactment than overall contemporaneous prevalence increases.^22^ In 2008 to 2016 yearly national survey data,^17^ among adults 26 years and older, the risk of adult CUD increased more after RCL enactment than before such enactment and more than overall national contemporaneous increases in prevalence. Neither CUD study used data after 2016, estimated the amount of overall change associated with the laws, or focused on a large health care population whose characteristics could increase vulnerability to effects of the changing laws.

The Veterans Health Administration (VHA) is the largest integrated health care system in the US,^23^ now providing care to over 6 million patients each year.^24^ The VHA patient population is predominantly male, has low income,^25,26^ and is characterized by high rates of psychiatric disorders^27^ and painful medical conditions incurred during military service.^26,28,29,30^ Compared with the general population and with other veterans, these characteristics increase the risk for CUD,^31,32^ making the VHA patient population a large, important group for studying the associations of MCLs and RCLs with outcomes. Additionally, many VHA patients are 65 years or older.^33^ Understanding CUD in older adults is important to inform screening and service planning given the disproportionately increasing rates of cannabis use in older adults.^34,35^ Therefore, leveraging the comprehensive VHA electronic health record, we investigated the role of MCLs and RCLs enactment in the national increases in rates of CUD diagnoses from 2005 to 2019, overall and by age group.

## Methods

Yearly data from 2005 to 2019 were obtained through the VHA Corporate Data Warehouse, a data repository for all care provided at VHA facilities or paid for by the VHA. Veterans aged 18 to 75 years with 1 or more VHA primary care, emergency department, or mental health visit in a given calendar year were included, except those in hospice/palliative care or residing outside the 50 states or Washington, DC. Resulting numbers ranging from 3 234 382 to 4 579 2994 patients each year were used to create 15 data sets, 1 for each year from 2005 to 2019. Waivers/exemptions of informed consent were granted by New York State Psychiatric Institute, VA Puget Sound, and VA New York Harbor Healthcare Systems institutional review boards.

### Measures

The primary outcome was a clinician-made CUD diagnosis given at 1 or more outpatient or inpatient encounters within a calendar year. *International Classification of Diseases, Ninth Revision, Clinical Modification (ICD-9-CM*) was used from 2005 to 2015 (305.2X, abuse; 304.3X, dependence). *International Statistical Classification of Diseases, Tenth Revision, Clinical Modification (ICD-10-CM) *was used from 2016 to 2019 (F12.1, abuse; F12.2, dependence). The abuse and dependence categories were combined because their criteria are unidimensional.^3^ Remission and unspecified cannabis use were excluded.

Primary exposures were state-year variables indicating state enactment of MCLs and/or RCLs, ie, that the law was operational and residents could rely on its legal protections. Patient state of residence was indicated by last health care encounter for each year. States were categorized each year as no cannabis laws (no CLs), MCLs only, and having MCLs/RCLs. Also, because state legal protection of dispensaries can occur post-MCL or -RCL enactment, potentially affecting availability, we used the RAND-USC Opioid Policy Tools and Information Center marijuana policy data^36^ to create state-year variables indicating the years that legally protected dispensaries were operational for medical cannabis in MCL-only states and for recreational cannabis in MCL/RCL states.

Individual control variables included age (continuous and categorized as 18-34, 35-64, and 65-75 years), sex (male/female), and race and ethnicity categories (Hispanic, non-Hispanic Black [hereafter, Black], non-Hispanic White [hereafter, White], other/multiple, and unknown), obtained from demographic files. Time-varying yearly state control covariates were state/year rates from American Community Survey data: percentage male, Black, Hispanic, White, 18 years or older, unemployed, income below poverty threshold, and yearly median household income. One-year estimates were used for 2005 to 2008,^37^ and 5-year estimates were used for 2009 to 2019,^38^ downloaded using the R tidycensus package (Tidycensus).^39^

### Statistical Analysis

Initial analyses of diagnosed CUD prevalence (hereafter, CUD prevalence) across 2005 to 2019 were grouped by state law status in 2019: (1) no CL, (2) MCL only, and (3) MCL/RCL. Adjusted prevalence estimates across each year in each of the 3 groups were obtained from a linear binomial regression model controlling for age, sex, race and ethnicity, and time-varying state covariates.

To estimate the role of MCL and RCL enactment in the national increases in CUD prevalence using all yearly information from 2005 to 2019, the staggered adoption difference-in-difference (DiD) model^40^ was used. This DiD model uses each state that enacts a law as its own control, comparing aggregated postlaw years to prelaw years and controlling for historical trends over time with data from all other states that had not enacted the respective law in contemporaneous years. A time-varying indicator was constructed for each state-year indicating no CL, MCL only, or MCL/RCL for that year. The DiD estimates for MCL only and MCL/RCL associations were obtained from fitting a linear binomial regression model with fixed effects for state, categorical year, time-varying law status, individual-level covariates, and time-varying state-level covariates. Resulting DiD estimates include the effect size of a state moving from no CLs to MCLs only and from MCLs only to MCLs/RCLs. (All states with RCLs previously had MCLs). The 17 no-CL states and 3 MCL-only states that did not change their laws between 2005 and 2019 contributed to DiD estimates by providing information for the contemporaneous trend estimates of the respective type of state. Note that the 2015 *ICD-9-CM* to *ICD-10-CM* change resulted in a slight downward shift in CUD prevalence in 2015 across the entire VHA system and all states.^13^ DiD estimates take this shift into account by using states that had not yet passed MCLs or RCLs, which experienced the *ICD-9-CM* to *ICD-10-CM* change at the same time, as contemporaneous secular controls. While 95% CIs are provided for DiD estimates, given the large numbers and resulting precise estimates, interpretation focuses on magnitude rather than *P* values. To illustrate the magnitude of the DiD estimates compared with the overall increases in CUD prevalence (ie, the amount of change that could be attributed to the laws), the DiD estimates were divided by the absolute changes between 2005 and 2019 in the states with the respective laws by 2019. To explore whether law outcomes differed by earlier or later enactment, we obtained state-specific DiD estimates with interaction terms between state and time-varying no-CL/MCL-only/RCL status. All procedures were then redone within age groups (18-34, 35-64, 65-75 years), adjusting for within-group continuous age.

Sensitivity analyses used similar methods. We examined legalized dispensaries by replacing state/year no-CL/MCL-only/RCL variables with the year medical or recreational dispensaries were first operational.^36^ We examined lagged MCL-only/RCL states effect sizes by replacing MCL-only/RCL state/year variables with 1-year postenactment dates. Analysis took place between February and December 2022.

## Results

### Demographic Characteristics

In 2005 (n = 3 234 382), 5.7%, 61.6%, and 32.7% of patients were aged 18 to 34, 35 to 64, and 65 to 75 years (eTable 1 in Supplement 1). In 2019 (n = 4 579 994), 10.5%, 48.8%, and 40.7% were in these age groups, respectively. Most patients were White (75.0% in 2005 and 66.6% in 2019) and male (94.1% in 2005 and 89.0% in 2019); female patients increased from 5.9% to 11.0% by 2019, as did Black and Hispanic patients (16.5% to 20.2%; 3.7% to 6.8%, respectively). The mean (SD) age was 57.0 [14.4] years.

### Trends in States Grouped by 2019 Cannabis Law Status

**Figure 1.**
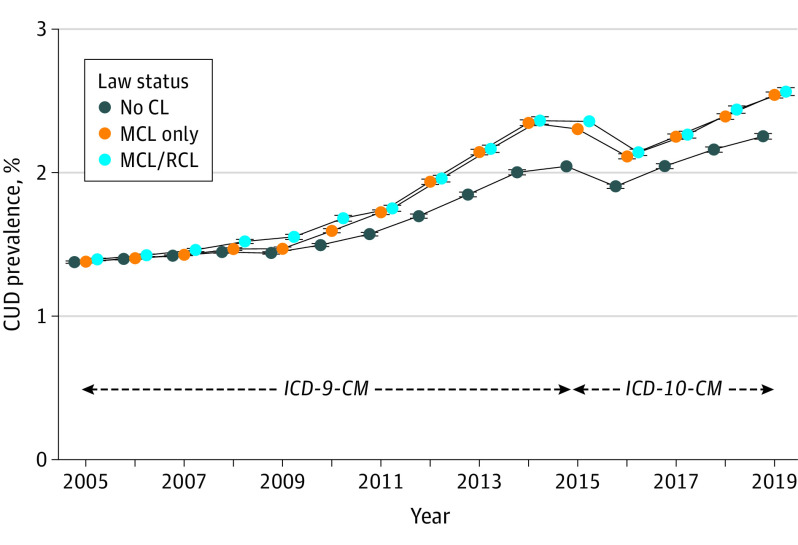
Trends in Prevalence of Cannabis Use Disorder (CUD) Diagnoses From 2005 to 2019, Aggregated Within the 3 Groups of States Defined by Their Cannabis Legalization Status at the End of 2019 Weighted mean prevalence estimates adjusted for age, sex, race and ethnicity, and time-varying state covariates are reported. For January 1, 2005, through September 30, 2015, *International Classification of Diseases, Ninth Revision, Clinical Modification* (*ICD-9-CM)* diagnostic codes were used; for October 1, 2015, through December 31, 2019, *International Statistical Classification of Diseases, Tenth Revision, Clinical Modification* (*ICD-10-CM*) codes were used. Note that estimates in 2015 represent a mix of diagnoses collected using *ICD-9-CM* and *ICD-10-CM* due to the systemwide transition on October 1, 2015. Error bars indicate 95% CIs; CIs are very small because of the large sample sizes. CL indicates cannabis law; MCL, medical cannabis law, RCL, recreational cannabis law.

Figure 1 and Table 1 show 2005 to 2019 CUD prevalence trends (weighted mean estimates) within no-CL, MCL-only, and MCL/RCL states defined by their 2019 status. In 2005 and 2019, CUD prevalence increased from 1.38% (95% CI, 1.37-1.38) to 2.25% (95% CI, 2.23-2.27) in no-CL states (0.88% absolute increase), from 1.38% (95% CI, 1.37-1.F) to 2.54% (95% CI, 2.52-2.56) in MCL-only states (1.16% absolute increase), and from 1.40% (95% CI, 1.39-1.40) to 2.56% (95% CI, 2.54-2.59) in MCL/RCL states (1.17% absolute increase; eTable 2 in Supplement 1). From 2005 to 2014, *ICD-9-CM* CUD prevalence increased in all 3 groups of states. As described previously,^13^ the *ICD-9-CM* to *ICD-10-CM* transition led to artifactual decreases in CUD prevalence in 2015 and 2016 due to clinician coding practices and electronic health record procedures in the VHA (eAppendix in Supplement 1). Thereafter, CUD prevalence resumed increases through 2019. eFigure 1A-C in Supplement 1 shows the corresponding estimates by age group. While the oldest group had the lowest prevalence and greatest relative increase over time, trends by state law status within age groups were similar to the overall sample.

**Table 1.** Adjusted CUD Prevalence in Veterans Health Administration Patients in 2005 and 2019, by Enacted State Law Status as of 2019, and Absolute Change Over Time

Type of state, by 2019	Overall			Age, y								
				18-34			35-64			65-75		
	CUD prevalence, %^a^		Absolute Change, %	CUD prevalence, %^b^		Absolute change, %	CUD prevalence, %^b^		Absolute change, %	CUD prevalence, %^b^		Absolute change, %
	2005	2019		2005	2019		2005	2019		2005	2019	
No CL (17 states)	1.38	2.25	0.88	1.41	4.51	3.10	1.24	2.80	1.57	0.35	0.94	0.59
MCL only (22 states)	1.38	2.54	1.16^c^	1.70	4.81	3.11^c^	1.38	3.38	1.99^c^	0.36	1.12	0.75^c^
MCL/RCL (11 states and Washington, DC)	1.39	2.56	1.17^d^	1.82	5.26	3.44^d^	1.59	3.45	1.86^d^	0.33	1.12	0.79^d^

Abbreviations: CL, cannabis law; CUD, cannabis use disorder; MCL, medical cannabis law; RCL, recreational cannabis law.

^a^ Adjusted for categorical age, sex, race and ethnicity, all age × race and ethnicity × sex interactions, yearly state-level median income, and yearly state rates of male individuals, Hispanic individuals, non-Hispanic Black individuals, non-Hispanic White individuals, those in the poverty category, those 18 years and older, and those who are unemployed.

^b^ Adjusted for continuous age, sex, race and ethnicity, all race and ethnicity × sex interactions, yearly state-level median income and yearly state rates of male individuals, Hispanic individuals, non-Hispanic Black individuals, non-Hispanic White individuals, those in the poverty category, those 18 years and older, and those who are unemployed.

^c^ Value used as denominator to determine the % of overall increase attributable to MCL enactment based on difference-in-difference estimates of MCL effect sizes.

^d^ Value used as denominator to determine the % of overall increase attributable to RCL enactment based on difference-in-difference estimates of RCL effect sizes.

### DiD Estimates of the Role of MCLs and RCLs in the National Increases in CUD Rates

The DiD estimate of the CUD prevalence increase due to MCL enactment was 0.05% (95% CI, 0.05%-0.06%; Table 2). Relative to the absolute change in MCL-only states by 2019 (1.16%; Table 1), 4.7% of this increase could be attributed to MCL enactment. The DiD estimate of the CUD prevalence increase due to changing from MCL only to RCL/MCL was 0.12% (95% CI, 0.10%-0.13%). Relative to the absolute change in MCL/RCL states by 2019 (1.17%; Table 1), 9.8% of the increase in RCL states could be attributed to RCL enactment.

**Table 2.** State MCL and RCL Enactment and Cannabis Use Disorder Prevalence in Veterans Health Administration Patients: DiD Estimates Using Data Across All Years, 2005-2019

Type of change in state law^a^	Overall			Age, y								
				18-34			35-64			65-75		
	Model-based DiD law result, % (95% CI)^b^	*P* value	% Of total absolute change accounted for by law change^c^	Model-based DiD law result, % (95% CI)^d^	*P* value	% Of total absolute change accounted for by law change^c^	Model-based DiD law result, % (95% CI)^d^	*P* value	% Of total absolute change accounted for by law change^c^	Model-based DiD law result, % (95% CI)^d^	*P* value	% Of total absolute change accounted for by law change^c^
No CL to MCL only	0.05 (0.05 to 0.06)	<.001	4.7	0.02 (−0.03 to 0.08)	.39	0.8	0.14 (0.12 to 0.16)	<.001	6.8	0.06 (0.05 to 0.07)	<.001	8.1
MCL only to RCL/MCL	0.12 (0.10 to 0.13)	<.001	9.8	0.002 (−0.07 to 0.08)	.95	0.1	0.05 (0.02 to 0.09)	<.001	2.9	0.15 (0.13 to 0.17)	<.001	18.6

Abbreviations: CL, cannabis law; DiD, difference-in-difference; MCL, medical cannabis law; RCL, recreational cannabis law.

^a^ A total of 22 states and Washington, DC, made a change from no CLs to MCLs only from 2005 to 2019; 11 states and Washington, DC, made a change from MCLs only to RCLs/MCLs during the period. Three of these states and Washington, DC, made both changes between 2005 and 2019 (ie, from no CLs to MCLs only and then later to RCLs/MCLs), hence contributing information to both associations. There were 20 states (3 with MCLs only and 17 with no CLs in 2019) that made no law changes between 2005 and 2019; in the DID model, they contribute to background secular trends. Model estimated effects represent the absolute increase (positive values) or decrease (negative values) in cannabis use disorder prevalence associated with law enactment. Confidence intervals not including 0.0 indicate significant changes. The DiD model compares the years after enactment (up to 2019 or until the next law change) in each state to the years before enactment (since 2005 or the previous law change) in the same state and controls for contemporaneous trends in other states that have not yet passed the respective law.

^b^ Staggered-adoption DID regression model,^40^ adjusted for categorical age, sex, race and ethnicity, all age × race and ethnicity × sex interactions, yearly state-level median income, and yearly state rates of male individuals, Hispanic individuals, non-Hispanic Black individuals, non-Hispanic White individuals, those in the poverty category, those 18 years and older, and those who are unemployed.

^c^ DiD estimate divided by absolute change across period as shown in Table 1

^d^ Staggered-adoption DID regression model,^40^ adjusted for continuous age, sex, race and ethnicity, all race and ethnicity × sex interactions, yearly state-level median income, and yearly state rates of male individuals, Black individuals, Hispanic individuals, White individuals, those in the poverty category, those 18 years and older, and those who are unemployed.

In patients aged 18 to 34 years (Table 2), neither MCL nor RCL enactment were significantly associated with the overall increase in CUD prevalence. In patients aged 35 to 64 years, in states enacting MCLs only, 6.8% of the increase in CUD prevalence could be attributed to MCLs, while in states enacting RCL, 2.9% of the increase in CUD prevalence could be attributed to RCLs. Among patients aged 65 to 75 years, in states enacting MCLs only, 8.1% of the increase in CUD prevalence was associated with MCLs, and in states enacting RCLs, 18.6% of the increase in CUD prevalence could be attributed to RCLs.

### State-Specific DiD Estimates

**Figure 2.**
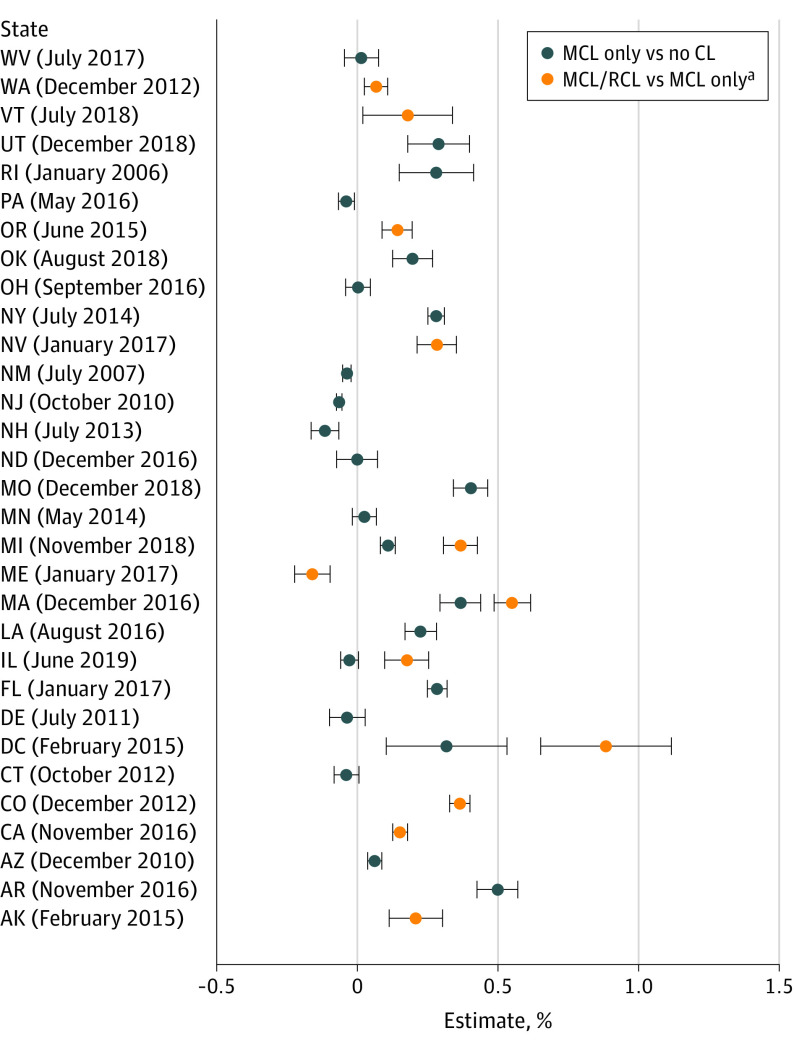
State-Specific Associations of Medical Cannabis Law (MCL) and Recreational Cannabis Law (RCL) Enactment With Cannabis Use Disorder Prevalence in Veterans Health Administration Patient for All Patients Aged 18 to 75 Years, Rank Ordered by the Month and Year the Most Recent Cannabis Law Was Enacted Point estimates and 95% CIs from the staggered-adoption difference-in-difference regression models are displayed. Estimated results represent the absolute increase (positive values) or decrease (negative values) in cannabis use disorder prevalence associated with cannabis law (CL) enactment. CIs not including 0.0 indicate significant changes. Note that 3 states and Washington, DC, changed from no CL to MCL only and then later MCL only to MCL/RCL between 2005 and 2019. Their dates of MCL enactment were as follows: Michigan, December 2008; Washington, DC, July 2010; Massachusetts, January 2013; and Illinois, January 2014. The MCL/RCL results shown here for Michigan, Washington, DC, Massachusetts, and Illinois are compared with no CL to facilitate comparison with the other MCL-only results shown. The estimate of the MCL/RCL vs MCL-only result is obtained by comparing the blue (MCL/RCL) to orange (MCL only) association in those states. ^a^In states that changed to MCL and RCL during the period, the MCL/RCL association plotted is compared with no CL for comparison with the MCL-only vs no-CL association.

Figure 2 shows the DiD estimates and 95% CIs for the 30 states and Washington, DC, that enacted MCLs and/or RCLs between 2005 and 2019, rank ordered by month and year of enactment (by RCLs if MCLs and RCLs were both enacted). eTable 3 in Supplement 1 shows 2005 and 2019 state-specific CUD prevalence and 95% CIs. Of the 22 states that changed from no CLs to MCLs only between 2005 and 2019, 11 showed an increase, 4 showed a decrease, and 7 showed no change associated with MCL enactment. Of the 11 states that enacted RCLs by 2019, 8 had MCLs before 2005. Of these, 7 showed increases in CUD prevalence associated with RCL enactment and 1, a decrease. Three states and Washington, DC, enacted both MCLs and RCLs between 2005 and 2019; 2 states showed increases associated with MCL enactment; and all showed increases associated with RCL enactment. Thus, of the 30 states that enacted MCLs, RCLs, or both between 2005 and 2019, 19 (63.3%) exhibited increased CUD associated with the laws. However, no state law association reached 1% absolute increase in CUD prevalence, and no patterning was evident by earlier or later enactment.

eFigure 2 in Supplement 1 shows state-specific DiD results by age. In patients aged 18 to 34 years, 12 states showed an increase associated with law enactment, while in those aged 35 to 64 years, 16 states showed an increase associated with law enactment. In patients aged 65 to 75 years, CUD prevalence increase was associated with MCLs or RCLs occurred in 19 of the 30 states and Washington, DC. However, few increases that could be attributable to state-specific MCL-only or RCL enactment were greater than 1% in magnitude, and no patterning was evident by earlier or later enactment.

### Sensitivity Analyses

Substituting operational dispensary dates for MCL only/RCL enactment dates, fewer states were analyzed because 4 MCL-only and 4 MCL/RCL states did not have operational dispensaries by 2019. This substitution produced a null effect in MCL-only states (95% CI, −0.02 to 0.003) but had little association with the positive result in MCL/RCL states (95% CI, 0.11%-0.15%) (eTables 4 and 5 in Supplement 1). Using 1-year postenactment lags did not meaningfully change results (eTables 6 and 7 in Supplement 1).

## Discussion

We examined the association of state medical and recreational cannabis legalization (MCLs and RCLs) to diagnosed CUD prevalence in VHA patients between 2005 and 2019, a period of increasing CUD prevalence in the US adult population and in VHA patients.^13^ DiD models that controlled for contemporaneous trends before and after MCL or RCL enactment in states with and without the respective laws suggested that CUD prevalence in VHA patients increased after MCL enactment by 0.05% more than would have occurred in the absence of MCLs and by an additional 0.12% more after RCL enactment than would have occurred in the absence of RCLs. These absolute increases represent the estimated effect sizes on CUD prevalence specifically associated with the enactment of the laws and imply that MCL and RCL accounted for 4.7% and 9.8%, respectively, of the 2005 to 2019 change in CUD prevalence. Considering states individually, a majority of MCL-enacting and RCL-enacting states had increases in CUD attributable with law enactments, although no state-specific increase attributable to law enactment reached 1%. By age, neither MCLs nor RCLs had a significant association with increases in CUD prevalence among those aged 18 to 34 years. While both MCL and RCL enactment were associated with CUD increases among patients aged 35 to 64 years, the largest effect size of RCLs was found in patients aged 65 to 75 years, in whom 8.1% of the increase in CUD prevalence could be attributed to MCL enactment, and 18.1% of the increase could be attributed to RCL enactment. Thus, in this national patient population, state cannabis legalization was followed by increases in CUD prevalence, but the increases attributable to the changing laws were relatively small compared with the overall increases in CUD prevalence.

Two US general population studies showed greater increases in adult CUD prevalence after states enacted cannabis laws relative to no-CL states.^17,22^ Between 1991 and 2013,^22^ CUD prevalence increased from 1.48% to 3.10% after MCL enactment, a 0.70% greater increase than contemporaneous increases in no-CL states. Between 2008 and 2016,^17^ CUD increased from 0.90% to 1.23% after RCL enactment among adults 26 years or older, a 0.33% greater percentage point increase than contemporaneous increases in no-CL states. Compared with the national survey results, VHA results are smaller (ie, 0.05% MCL; 0.12% MCL/RCL, summing to 0.17%, a global estimate of effect sizes associated with change from no CL to RCL) but similar to the general population studies in showing a significant although modest role of MCL and RCL in the national increases in CUD prevalence. The lack of RCL results among young adults in the general population study,^17^ together with our null results in patients aged 18 to 34 years, suggests that MCLs and RCLs operate differently among younger and older individuals. Possible explanations for these age differences include that younger individuals may be less concerned about the legal status of cannabis than older individuals, who may be more law-abiding or less risk-taking, or that younger individuals have readier access to illicit cannabis, making legalization less relevant to whether they use cannabis and subsequently, in a vulnerable subset, develop CUD.

In sensitivity analyses, 1-year lags did not meaningfully change results, nor did replacing RCL enactment year with the year recreational dispensaries became operational. However, replacing MCL enactment year with the year medical dispensaries became operational eliminated a result for MCL-only states. This suggests that MCL enactment may work through influences on perceived safety rather than through greater distribution and availability via medical dispensaries.

Factors that potentially minimized our estimates of the role of MCL and RCL in the national increases in CUD prevalence include a general diffusion of positive attitudes toward cannabis use, decreased harm perception, and increased use across the entire US adult population as more and more states legalized medical and recreational cannabis use. Perceived risk has decreased,^14,15^ and despite inconclusive evidence, a majority of adults now see cannabis as beneficial to treat or prevent health problems.^41^ The multibillion dollar cannabis industry,^42^ seeking further expansion, must increase demand by generating new customers and/or by generating greater use among existing ones.^43^ Websites of medical cannabis companies often imply product safety and efficacy, potentially leading policy makers and the public into believing unconfirmed claims.^44,45^ Cannabis companies carefully design social media promotional profiles to attract customers^45^ via content focusing on cannabis normalization,^46^ and state regulations of cannabis advertising are often violated.^45,47,48^ These industry activities could contribute to changing attitudes, increased cannabis use, and CUD among some users, both within and across state boundaries.

Additional possible mechanisms of the overall increases in CUD prevalence that should be examined in future studies include increasing rates of CUD risk factors, eg, pain^49,50^ and psychiatric disorders,^51^ and the increasing tetrahydrocannabinol (THC) potency of cannabis,^16^ which increases addiction potential.^52,53^ Highly potent cannabis products are increasingly popular in both medical and recreational cannabis markets.^54,55,56,57^

### Limitations

Study limitations are noted. First, VHA patients are not representative of all veterans^25,30^ or all adults. Second, the *ICD* CUD diagnoses were made by clinicians, not structured research assessments. VHA clinicians are most likely to diagnose severe disorders^58,59^ and may miss the mild cases commonly found in general population surveys using structured assessment instruments.^9,31^ While the actual number of missed VHA cases is unknown and may have varied over time, the overall 2019 VHA CUD prevalence (1.9%)^60^ was higher than in National Survey on Drug Use and Health adults (1.7%),^61^ despite the younger mean age of adult National Survey on Drug Use and Health participants. Thus, VHA findings serve as a useful counterpart to general population findings, providing information on what are likely to be severe cases in a clinical population in a national health care system that has many risk factors for CUD. Further, research diagnostic interviews with 3 to 4 million patients yearly are not feasible, so the VHA data provide a unique opportunity to examine the role of MCL and RCL in the national increases in CUD prevalence. Third, cannabis law provisions are heterogeneous.^62,63,64^ We examined whether states permitted dispensaries, but other differences (eg, possession limits, price, taxation) should be addressed in future studies. Fourth, state law effects may be delayed. We analyzed 1-year lags to include recently enacted RCLs; longer lags should be analyzed later. Fifth, demographic characteristics could influence vulnerability to MCLs or RCLs; studies should investigate these as modifiers of law effects. Sixth, a cannabis use measure was not available, so CUD within users and specific use patterns could not be examined. Seventh, while we controlled for many time-varying state-level confounders, others (eg, state opioid policies) could have affected results and should be addressed in future studies. Eighth, the DiD methodology estimates law effects in the states that enacted them and does not account for spillover effects into other states. If patients in no-CL states (the contemporaneous secular controls) are influenced by CLs in other states, estimated CLs effects will be biased toward the null. For example, border crossing to buy cannabis by patients in no-CL states living near a border with an MCL-only or RCL state^65^ could have elevated CUD rates in some no-CL states, potentially mitigating the ability to find stronger MCL or RCL effects at the state level. Ninth, no study can be unequivocal about the causal nature of observed effects. However, our pre-post DiD analysis that controlled for contemporaneous trends and many other state-level factors that might have changed over time provides stronger support for estimated effects of changing cannabis laws than studies without such design and analytic rigor.^66,67,68,69^ These limitations are offset by notable strengths and novel aspects, including a large sample size, information about MCL/RCL associations in an important older age group about whom little is known, estimates of state-specific MCL and RCL results by the order (year) in which they were enacted, and a focus not only on significance but also on the magnitude of effect sizes.

## Conclusions

In this study, cannabis did not have the same overdose/mortality risk profile as opioids or stimulants. However, CUD, a diagnosable disorder with many associated problems,^5,6,7,8,70^ is prevalent among cannabis users (approximately 20%-33%), which is more than commonly assumed.^2^ The US national increase in CUD diagnoses regardless of state laws underscores a growing need in the VHA and elsewhere to screen for cannabis use and offer evidence-based treatments for CUD.^71^ Additionally, while VHA patients aged 65 to 75 years had the lowest CUD prevalence in this study, estimated RCL results were strongest in this group, suggesting a need to attend to potential CUD in older veterans.

To conclude, alcohol, tobacco, and prescription opioids have undergone major shifts in public acceptance or rejection across decades and generations.^72,73^ Public health efforts regarding these substances have long competed with commercial interests. With cannabis increasingly legalized, similar competing public health and commercial interests are now emerging. To inform future health and policy efforts, researchers must monitor harms related to increasing CUD, identify whether subgroups show particular risk due to changing cannabis laws, and ensure that this knowledge is clearly communicated to policy makers, clinicians, and the public.

## Notes

Supplement 1. eTable 1. Demographic characteristics of the VHA patient population in 2005 and 2019 eTable 2. CUD prevalence (weighted mean estimates) from 2005 to 2019, aggregated within the three groups of states defined by their status at the end of 2019, overall and stratified by age group eTable 3. CUD prevalence, weighted mean estimates at 2005 and 2019, by state eTable 4. Adjusted CUD prevalence in VHA patients in 2005 and 2019 by whether states permitted legal operation of dispensaries, and absolute change over time eTable 5. Effects of changes due to whether states legally permitted dispensaries on CUD prevalence in VHA patients: Difference-in-difference (DiD) estimates incorporating data across all years 2005 – 2019 eTable 6. Adjusted CUD prevalence in VHA patients in 2005 and 2019 by enacted state law status with 1-year lag, and absolute change over time eTable 7. Effects of changes due to state MCL and RCL enactment plus a 1-year lag on CUD prevalence in VHA patients incorporating data across all years 2005 – 2019: Difference-in-difference (DiD) estimates eFigure 1. Trends in prevalence of CUD diagnoses from 2005 to 2019 (adjusted weighted mean estimates), aggregated within the three groups of states defined by their cannabis legalization status at the end of 2019, stratified by age eFigure 2. State-specific effects of MCL and RCL enactment on CUD prevalence in VHA patients stratified by age eAppendix. eReferences

Supplement 2. Data sharing statement
