# Supplementary material for: State Cannabis Legalization and Cannabis Use Disorder in the US Veterans Health Administration, 2005 to 2019
Source: JAMA Psychiatry. 2023 Mar 1;80(4):380–8. doi: 10.1001/jamapsychiatry.2023.0019 (PMC9979011; doi:10.1001/jamapsychiatry.2023.0019)
Supplement: Supplement 1. — eTable 1. Demographic characteristics of the VHA patient population in 2005 and 2019 eTable 2. CUD prevalence (weighted mean estimates) from 2005 to 2019, aggregated within the three groups of states defined by their status at the end of 2019, overall and stratified by age group eTable 3. CUD prevalence, weighted mean estimates at 2005 and 2019, by state eTable 4. Adjusted CUD prevalence in VHA patients in 2005 and 2019 by whether states permitted legal operation of dispensaries, and absolute change over time eTable 5. Effects of changes due to whether states legally permitted dispensaries on CUD prevalence in VHA patients: Difference-in-difference (DiD) estimates incorporating data across all years 2005 – 2019 eTable 6. Adjusted CUD prevalence in VHA patients in 2005 and 2019 by enacted state law status with 1-year lag, and absolute change over time eTable 7. Effects of changes due to state MCL and RCL enactment plus a 1-year lag on CUD prevalence in VHA patients incorporating data across all years 2005 – 2019: Difference-in-difference (DiD) estimates eFigure 1. Trends in prevalence of CUD diagnoses from 2005 to 2019 (adjusted weighted mean estimates), aggregated within the three groups of states defined by their cannabis legalization status at the end of 2019, stratified by age eFigure 2. State-specific effects of MCL and RCL enactment on CUD prevalence in VHA patients stratified by age eAppendix. eReferences [file jamapsychiatry-e230019-s001.pdf]

Supplemental Online Content

Hasin DS, Wall MM, Choi CJ, et al. State cannabis legalization and cannabis use disorder in the US Veterans Health Administration, 2005 to 2019. *JAMA Psychiatry*. Published online March 1, 2023. doi:10.1001/jamapsychiatry.2023.0019

eTable 1. Demographic characteristics of the VHA patient population in 2005 and 2019
eTable 2. CUD prevalence (weighted mean estimates) from 2005 to 2019, aggregated within the three groups of states defined by their status at the end of 2019, overall and stratified by age group
eTable 3. CUD prevalence, weighted mean estimates at 2005 and 2019, by state
eTable 4. Adjusted CUD prevalence in VHA patients in 2005 and 2019 by whether states permitted legal operation of dispensaries, and absolute change over time
eTable 5. Effects of changes due to whether states legally permitted dispensaries on CUD prevalence in VHA patients: Difference-in-difference (DiD) estimates incorporating data across all years 2005 – 2019
eTable 6. Adjusted CUD prevalence in VHA patients in 2005 and 2019 by enacted state law status with 1-year lag, and absolute change over time
eTable 7. Effects of changes due to state MCL and RCL enactment plus a 1-year lag on CUD prevalence in VHA patients incorporating data across all years 2005 – 2019: Difference-in-difference (DiD) estimates
eFigure 1. Trends in prevalence of CUD diagnoses from 2005 to 2019 (adjusted weighted mean estimates), aggregated within the three groups of states defined by their cannabis legalization status at the end of 2019, stratified by age
eFigure 2. State-specific effects of MCL and RCL enactment on CUD prevalence in VHA patients stratified by age
eAppendix.
eReferences

This supplemental material has been provided by the authors to give readers additional information about their work.

© 2023 Hasin DS et al. *JAMA Psychiatry.*

eTable 1. Demographic characteristics of the VHA patient population in 2005 and 2019

	2005 (N=3,234,382)		2019 (N=4,579,994)		SMD [a]	P [b]
	n	% or Mean (SD)	n	% or Mean (SD)		
Age (continuous)	3,234,382	57.9 (12.1)	4,579,994	57.0 (14.4)	0.069	<.001
Age (categorical)						<.001
18-34	182,809	5.7%	482,493	10.5%	0.627	
35-64	1,993,492	61.6%	2,235,435	48.8%	0.517	
65-75	1,058,081	32.7%	1,862,066	40.7%	0.339	
Sex						<.001
Female	191,146	5.9%	504,244	11.0%	0.629	
Male	3,043,236	94.1%	4,075,750	89.0%	0.629	
Race/Ethnicity						<.001
AmInd/AlaskNative	18,252	0.6%	34,529	0.8%	0.283	
Asian	17,224	0.5%	55,256	1.2%	0.733	
Black	533,809	16.5%	926,783	20.2%	0.246	
Hispanic/Latino	120,569	3.7%	311,161	6.8%	0.587	
MultipleRace/Eth	22,744	0.7%	41,312	0.9%	0.245	
PacIs/NatHawaiian	22,873	0.7%	33,232	0.7%	0.026	
Unknown	72,939	2.3%	128,250	2.8%	0.217	
White	2,425,972	75.0%	3,049,471	66.6%	0.402	

[a]Standardized absolute mean difference is calculated as the average absolute difference between groups divided by the overall standard deviation.

[b]p-values for t-test of continuous variables and chi-square tests of categorical variables.

© 2023 Hasin DS et al. *JAMA Psychiatry.*

eTable 2. CUD prevalence (weighted mean estimates) from 2005 to 2019, aggregated within the three groups of states defined by their status at the end of 2019, overall and stratified by age group			
	**No-CL**	**MCL-Only**	**MCL/RCL**
**Year**	**% (95% CI)**	**% (95% CI)**	**% (95% CI)**
	**Overall**		
2005	1.376 (1.368, 1.384)	1.378 (1.370, 1.386)	1.395 (1.386, 1.404)
2006	1.398 (1.391, 1.405)	1.403 (1.397, 1.410)	1.423 (1.415, 1.432)
2007	1.420 (1.413, 1.426)	1.428 (1.421, 1.434)	1.461 (1.450, 1.472)
2008	1.446 (1.438, 1.453)	1.467 (1.458, 1.476)	1.520 (1.506, 1.534)
2009	1.440 (1.432, 1.448)	1.470 (1.459, 1.481)	1.551 (1.533, 1.570)
2010	1.496 (1.485, 1.506)	1.594 (1.580, 1.609)	1.682 (1.661, 1.702)
2011	1.571 (1.558, 1.583)	1.723 (1.708, 1.739)	1.751 (1.730, 1.772)
2012	1.696 (1.682, 1.711)	1.936 (1.918, 1.954)	1.958 (1.934, 1.981)
2013	1.847 (1.831, 1.864)	2.143 (2.123, 2.162)	2.165 (2.140, 2.191)
2014	2.002 (1.984, 2.020)	2.347 (2.326, 2.368)	2.363 (2.336, 2.389)
2015	2.045 (2.027, 2.063)	2.356 (2.336, 2.376)	2.302 (2.277, 2.327)
2016	1.904 (1.889, 1.920)	2.113 (2.096, 2.130)	2.140 (2.118, 2.163)
2017	2.044 (2.027, 2.062)	2.250 (2.232, 2.269)	2.264 (2.240, 2.288)
2018	2.160 (2.142, 2.178)	2.391 (2.371, 2.410)	2.438 (2.413, 2.464)
2019	2.252 (2.233, 2.271)	2.540 (2.520, 2.561)	2.564 (2.537, 2.591)
	**Age 18-34**		
2005	1.411 (1.330, 1.492)	1.703 (1.601, 1.806)	1.819 (1.721, 1.917)
2006	1.604 (1.535, 1.674)	1.918 (1.831, 2.004)	2.129 (2.038, 2.220)
2007	1.738 (1.670, 1.806)	2.081 (2.012, 2.150)	2.326 (2.237, 2.416)
2008	2.054 (1.985, 2.122)	2.308 (2.229, 2.387)	2.596 (2.507, 2.685)
2009	2.224 (2.155, 2.292)	2.502 (2.409, 2.595)	2.687 (2.577, 2.797)
2010	2.447 (2.358, 2.535)	3.007 (2.912, 3.102)	3.044 (2.941, 3.146)
2011	2.625 (2.542, 2.708)	3.255 (3.161, 3.348)	3.195 (3.092, 3.299)
2012	2.986 (2.902, 3.071)	3.605 (3.511, 3.700)	3.605 (3.494, 3.716)
2013	3.374 (3.287, 3.462)	4.061 (3.963, 4.159)	3.924 (3.809, 4.039)
2014	3.679 (3.591, 3.766)	4.349 (4.252, 4.446)	4.221 (4.108, 4.334)
2015	3.847 (3.759, 3.935)	4.472 (4.377, 4.568)	4.168 (4.062, 4.275)
2016	3.776 (3.690, 3.862)	4.226 (4.133, 4.319)	4.149 (4.045, 4.254)
2017	4.152 (4.060, 4.244)	4.455 (4.357, 4.553)	4.560 (4.448, 4.673)
2018	4.379 (4.282, 4.476)	4.656 (4.552, 4.760)	4.898 (4.777, 5.019)
2019	4.515 (4.414, 4.615)	4.814 (4.706, 4.922)	5.259 (5.129, 5.389)
	**Age 35-64**		
2005	1.235 (1.212, 1.259)	1.383 (1.351, 1.416)	1.589 (1.560, 1.619)
2006	1.424 (1.404, 1.443)	1.698 (1.673, 1.724)	1.886 (1.864, 1.907)
2007	1.537 (1.515, 1.559)	1.772 (1.747, 1.797)	1.994 (1.959, 2.030)
2008	1.670 (1.649, 1.690)	1.977 (1.957, 1.998)	2.184 (2.147, 2.220)
2009	1.719 (1.698, 1.739)	2.013 (1.982, 2.043)	2.202 (2.160, 2.244)
2010	1.882 (1.854, 1.909)	2.291 (2.260, 2.321)	2.403 (2.364, 2.442)
2011	2.059 (2.030, 2.087)	2.477 (2.446, 2.509)	2.487 (2.446, 2.528)
2012	2.268 (2.238, 2.298)	2.799 (2.764, 2.833)	2.729 (2.683, 2.774)
2013	2.437 (2.405, 2.470)	3.064 (3.027, 3.101)	2.971 (2.922, 3.020)
2014	2.621 (2.587, 2.654)	3.360 (3.321, 3.399)	3.196 (3.145, 3.246)
2015	2.612 (2.579, 2.645)	3.305 (3.266, 3.344)	3.053 (3.005, 3.102)
2016	2.323 (2.293, 2.353)	2.847 (2.811, 2.882)	2.812 (2.767, 2.857)
2017	2.542 (2.510, 2.574)	3.022 (2.985, 3.059)	3.035 (2.987, 3.082)
2018	2.672 (2.639, 2.706)	3.212 (3.174, 3.251)	3.297 (3.247, 3.347)
2019	2.805 (2.770, 2.839)	3.378 (3.338, 3.418)	3.447 (3.394, 3.499)
	**Age 65-75**		
2005	0.352 (0.346, 0.359)	0.361 (0.353, 0.369)	0.327 (0.316, 0.339)

© 2023 Hasin DS et al. *JAMA Psychiatry.*

2006	0.359 (0.353, 0.366)	0.348 (0.338, 0.358)	0.334 (0.327, 0.341)
2007	0.360 (0.354, 0.366)	0.359 (0.352, 0.366)	0.357 (0.351, 0.364)
2008	0.358 (0.352, 0.364)	0.374 (0.365, 0.383)	0.336 (0.330, 0.342)
2009	0.325 (0.318, 0.331)	0.340 (0.333, 0.347)	0.293 (0.285, 0.302)
2010	0.339 (0.333, 0.344)	0.361 (0.355, 0.368)	0.346 (0.333, 0.358)
2011	0.349 (0.343, 0.356)	0.346 (0.337, 0.354)	0.356 (0.336, 0.377)
2012	0.355 (0.341, 0.368)	0.434 (0.417, 0.451)	0.499 (0.472, 0.525)
2013	0.455 (0.437, 0.473)	0.574 (0.555, 0.594)	0.672 (0.643, 0.701)
2014	0.582 (0.562, 0.602)	0.737 (0.716, 0.758)	0.858 (0.827, 0.888)
2015	0.658 (0.637, 0.679)	0.799 (0.778, 0.820)	0.863 (0.834, 0.893)
2016	0.596 (0.578, 0.615)	0.699 (0.680, 0.717)	0.761 (0.735, 0.787)
2017	0.720 (0.700, 0.741)	0.836 (0.816, 0.855)	0.850 (0.823, 0.877)
2018	0.848 (0.826, 0.871)	0.967 (0.945, 0.988)	0.999 (0.970, 1.029)
2019	0.944 (0.921, 0.968)	1.115 (1.092, 1.139)	1.118 (1.087, 1.150)

© 2023 Hasin DS et al. *JAMA Psychiatry.*

eTable 3. CUD prevalence, weighted mean estimates at 2005 and 2019, by state.		
	**CUD Prevalence**	
**State**	**2005<br>% (95% CI)**	**2019<br>% (95% CI)**
AK	1.502 (0.681, 2.323)	2.933 (1.371, 4.495)
AL	1.479 (0.354, 2.603)	2.226 (0.643, 3.809)
AR	1.970 (0.464, 3.477)	3.585 (0.953, 6.216)
AZ	1.478 (0.356, 2.599)	2.153 (0.765, 3.541)
CA	1.616 (0.644, 2.588)	2.439 (0.919, 3.959)
CO	1.553 (0.423, 2.683)	2.744 (1.060, 4.427)
CT	1.008 (0.001, 2.333)	2.077 (0.520, 3.633)
DC	1.461 (0.001, 3.061)	2.621 (0.802, 4.441)
DE	0.802 (0.060, 1.543)	2.399 (0.255, 4.544)
FL	1.482 (0.345, 2.618)	2.857 (1.180, 4.534)
GA	1.307 (0.565, 2.049)	2.320 (0.516, 4.124)
HI	2.278 (0.525, 4.031)	2.283 (1.168, 3.399)
IA	1.009 (0.001, 2.097)	2.251 (0.461, 4.041)
ID	1.071 (0.001, 2.326)	1.807 (0.530, 3.084)
IL	1.135 (0.293, 1.978)	2.423 (0.493, 4.354)
IN	1.207 (0.001, 2.556)	2.259 (0.369, 4.148)
KS	1.055 (0.001, 2.137)	2.709 (0.835, 4.584)
KY	1.115 (0.197, 2.034)	2.217 (0.422, 4.011)
LA	1.204 (0.296, 2.113)	2.751 (0.820, 4.682)
MA	1.318 (0.001, 2.656)	2.903 (0.664, 5.142)
MD	1.101 (0.161, 2.041)	1.781 (0.774, 2.788)
ME	1.226 (0.179, 2.272)	1.571 (0.440, 2.703)
MI	1.357 (0.321, 2.394)	2.591 (0.478, 4.704)
MN	1.740 (0.138, 3.342)	2.038 (0.158, 3.919)
MO	1.284 (0.301, 2.268)	2.583 (0.715, 4.451)
MS	1.080 (0.000, 2.165)	2.418 (0.788, 4.047)
MT	0.905 (0.152, 1.657)	1.597 (0.493, 2.702)
NC	1.057 (0.219, 1.895)	2.114 (0.549, 3.680)
ND	0.830 (0.043, 1.617)	1.702 (0.372, 3.032)
NE	1.077 (0.001, 2.386)	1.728 (0.327, 3.128)
NH	0.872 (0.001, 1.870)	1.677 (0.386, 2.969)
NJ	0.578 (0.001, 1.962)	1.720 (0.304, 3.136)
NM	1.139 (0.001, 2.533)	1.822 (0.561, 3.083)
NV	1.301 (0.411, 2.191)	2.831 (1.058, 4.605)
NY	1.383 (0.269, 2.497)	2.508 (0.671, 4.345)
OH	2.405 (0.467, 4.344)	3.131 (0.855, 5.406)
OK	1.388 (0.158, 2.619)	2.461 (0.820, 4.102)
OR	2.019 (0.540, 3.498)	2.635 (0.791, 4.478)
PA	1.015 (0.001, 2.067)	2.063 (0.165, 3.961)
RI	1.623 (0.233, 3.013)	2.793 (0.827, 4.759)
SC	1.024 (0.215, 1.832)	1.887 (0.525, 3.249)
SD	1.130 (0.001, 2.300)	1.770 (0.513, 3.026)

© 2023 Hasin DS et al. *JAMA Psychiatry*.

TN	1.106 (0.016, 2.197)	2.451 (0.763, 4.140)
TX	1.586 (0.550, 2.622)	2.442 (0.965, 3.920)
UT	1.200 (0.001, 2.409)	2.130 (0.773, 3.488)
VA	1.203 (0.417, 1.990)	2.044 (0.850, 3.237)
VT	0.949 (0.001, 2.058)	2.021 (0.232, 3.810)
WA	2.095 (0.764, 3.427)	2.235 (0.972, 3.499)
WI	1.178 (0.127, 2.228)	1.916 (0.409, 3.424)
WV	1.101 (0.310, 1.892)	2.032 (0.127, 3.937)
WY	1.032 (0.009, 2.055)	1.755 (0.421, 3.088)

© 2023 Hasin DS et al. *JAMA Psychiatry*.

eTable 4. Adjusted CUD prevalence in VHA patients in 2005 and 2019 by whether states permitted legal operation of dispensaries, and absolute change over time												
Type of State	CUD prevalence[a] %		Absolute Change	CUD prevalence[b]		Absolute Change %	CUD prevalence[b]		Absolute Change %	CUD prevalence[b]		Absolute Change %
	2005	2019	%	2005	2019	%	2005	2019	%	2005	2019	%
	Overall			Age 18-34			Age 35-64			Age 65-75		
No dispensaries by 2019 (17 no-CL, 4 MCL-only states)	1.34	2.26	0.92	1.29	4.50	3.21	1.09	2.79	1.69	0.34	0.95	0.61
Medical but not recreational dispensary by 2019 (18 MCL-only states, 3 MCL/RCL states and DC)	1.35	2.53	1.18	1.58	4.87	3.30	1.31	3.39	2.08	0.34	1.10	0.76
Recreational dispensary by 2019 (8 MCL/RCL states)	1.39	2.62	1.23	1.80	5.39	3.59	1.63	3.57	1.94	0.32	1.17	0.85

[a] Adjusted for categorical age, sex, race/ethnicity, all age*race/ethnicity*sex interactions, yearly state-level median income, and yearly state rates of males, Hispanics, non-Hispanic Whites, non-Hispanic Blacks, those in poverty, those age 18+, and those unemployed..
[b] Adjusted for continuous age, sex, race/ethnicity, all race/ethnicity*sex interactions, yearly state-level median income, and yearly state rates of males, Hispanics, non-Hispanic Whites, non-Hispanic Blacks, those in poverty, those age 18+, and those unemployed..

© 2023 Hasin DS et al. *JAMA Psychiatry.*

eTable 5. Effects of changes due to whether states legally permitted dispensaries on CUD prevalence in VHA patients: Difference-in-difference (DiD) estimates incorporating data across all years 2005 – 2019								
	Model-based DiD law effect [a] % p-value 95% CI	% of absolute change attributable to law [b]	Model-based DiD law effect [c] % p-value 95% CI	% of absolute change attributable to law [b]	Model-based DiD law effect [c] % p-value 95% CI	% of absolute change attributable to law [b]	Model-based DiD law effect [c] % p-value 95% CI	% of absolute change attributable to law [b]
	**Overall**		**Age 18-34**		**Age 35-64**		**Age 65-75**	
Effect of change to medical dispensaries[d]	-0.008 (0.144) -0.019, 0.003	-0.7%	-0.195 (<.001) -0.250, -0.141	-5.9%	-0.010 (0.363) -0.031, 0.011	-0.5%	0.014 (0.013) 0.003, 0.025	1.9%
Effect of change to recreational dispensaries[d]	0.127 (<.001) 0.107, 0.147	10.3%	0.227 (<.001) 0.138, 0.315	6.3%	0.091 (<.001) 0.055, 0.127	4.7%	0.145 (<.001) 0.123, 0.168	17.2%

[a] Staggered-Adoption Difference-in-Difference (DID) model[1] adjusted for categorical age, sex, race/ethnicity, all age*race/ethnicity*sex interactions, yearly state-level median income, and yearly state rates of males, Hispanics, non-Hispanic Whites, non-Hispanic Blacks, those in poverty, those age 18+, and those unemployed..

[b] DiD estimate divided by absolute change across period (see E-Table 4)

[c] Staggered-Adoption Difference-in-Difference (DID) model[1] adjusted for continuous age, sex, race/ethnicity, all race/ethnicity*sex interactions, yearly state-level median income, and yearly state rates of males, Hispanics, non-Hispanic Whites, non-Hispanic Blacks, those in poverty, those age 18+, and those unemployed.

[d] 21 states and DC made a change to having medical dispensaries during the period; 2 states made a change to having recreational dispensaries and 6 states made a change to medical and to recreational dispensaries

© 2023 Hasin DS et al. *JAMA Psychiatry.*

eTable 6. Adjusted CUD prevalence in VHA patients in 2005 and 2019 by enacted state law status with 1-year lag, and absolute change over time

	CUD prevalence[a] %		Absolute Change	CUD prevalence[b] %		Absolute Change	CUD prevalence[b] %		Absolute Change	CUD prevalence[b] %		Absolute Change
Type of State	2005	2019	%	2005	2019	%	2005	2019	%	2005	2019	%
	Overall			Age 18-34			Age 35-64			Age 65-75		
No-CL (17 states by 2019)	1.36	2.25	0.89	1.35	4.51	3.15	1.09	2.65	1.56	0.34	0.94	0.59
MCL-Only (23 states by 2019)[c]	1.36	2.54	1.18	1.61	4.86	3.25	1.38	3.36	1.98	0.35	1.10	0.75
MCL/RCL (10 states and DC by 2019)[c]	1.39	2.59	1.20	1.82	5.30	3.48	1.56	3.71	2.15	0.33	1.14	0.81

[a] Adjusted for categorical age, sex, race/ethnicity, all age*race/ethnicity*sex interactions, yearly state-level median income, and yearly state rates of males, Hispanics, non-Hispanic Whites, non-Hispanic Blacks, those in poverty, those age 18+, and those unemployed.

[b] Adjusted for continuous age, sex, race/ethnicity, all race/ethnicity*sex interactions, yearly state-level median income, and yearly state rates of males, Hispanics, non-Hispanic Whites, non-Hispanic Blacks, those in poverty, those age 18+, and those unemployed..

[c] MCL-only and MCL/RCL states differ from Table 2 by 1 because Illinois (IL) which enacted RCL in 2019 is considered a MCL-only state here to account for the 1-year lag

© 2023 Hasin DS et al. *JAMA Psychiatry.*

eTable 7. Effects of changes due to state MCL and RCL enactment plus a 1-year lag on CUD prevalence in VHA patients incorporating data across all years 2005 – 2019: Difference-in-difference (DiD) estimates

	Model-based DiD law effect [a] % p-value 95% CI	% of absolute change attributable to law [c]	Model-based DiD law effect [b] % p-value 95% CI	% of absolute change attributable to law [c]	Model-based DiD law effect [b] % p-value 95% CI	% of absolute change attributable to law [c]	Model-based DiD law effect [b] % p-value 95% CI	% of absolute change attributable to law [c]
	Overall		Age 18-34		Age 35-64		Age 65-75	
Effect of change from no-CL to MCL-only[d]	0.046 (<.001) 0.035, 0.056	3.9%	-0.016 (0.588) -0.075, 0.043	-0.5%	0.123 (<.001) 0.101, 0.145	6.2%	0.032 (<.001) 0.024, 0.041	4.3%
Effect of change from MCL-only to RCL[d]	0.128 (<.001) 0.109, 0.146	10.7%	0.135 (0.001) 0.052, 0.218	3.9%	0.076 (<.001) 0.042, 0.110	3.5%	0.151 (<.001) 0.130, 0.172	18.7%

[a] Staggered-Adoption Difference-in-Difference (DID) model[1] adjusted for categorical age, sex, race/ethnicity, all age*race/ethnicity*sex interactions, yearly state-level median income, and yearly state rates of males, Hispanics, non-Hispanic Whites, non-Hispanic Blacks, those in poverty, those age 18+, and those unemployed..

[b] Staggered-Adoption Difference-in-Difference (DID) model[1] adjusted for continuous age, sex, race/ethnicity, all race/ethnicity*sex interactions, yearly state-level median income, and yearly state rates of males, Hispanics, non-Hispanic Whites, non-Hispanic Blacks, those in poverty, those age 18+, and those unemployed..

[c] DiD estimate divided by absolute change across period (see E-Table 6)

[d] 22 states and DC made a change from no-CL to MCL-only during the period from 2005-2018 (to account for the 1-year lag); 10 states and DC made a change from MCL-only to RCL/MCL during the period. Note, 2 of these states and DC made both changes during the period from no-CL to MCL-only and then to RCL/MCL hence contribute to both effects. With the 1-year lag, 20 states (3 with MCL-only and 17 with no-CL) made no law changes between 2005-2019 and contribute in the DID model to background secular trends.

© 2023 Hasin DS et al. *JAMA Psychiatry.*

eFigure 1a. Trends in prevalence of CUD diagnoses from 2005 to 2019 (adjusted weighted mean estimates[a]), aggregated within the three groups of states defined by their cannabis legalization status at the end of 2019: Patients age 18-34

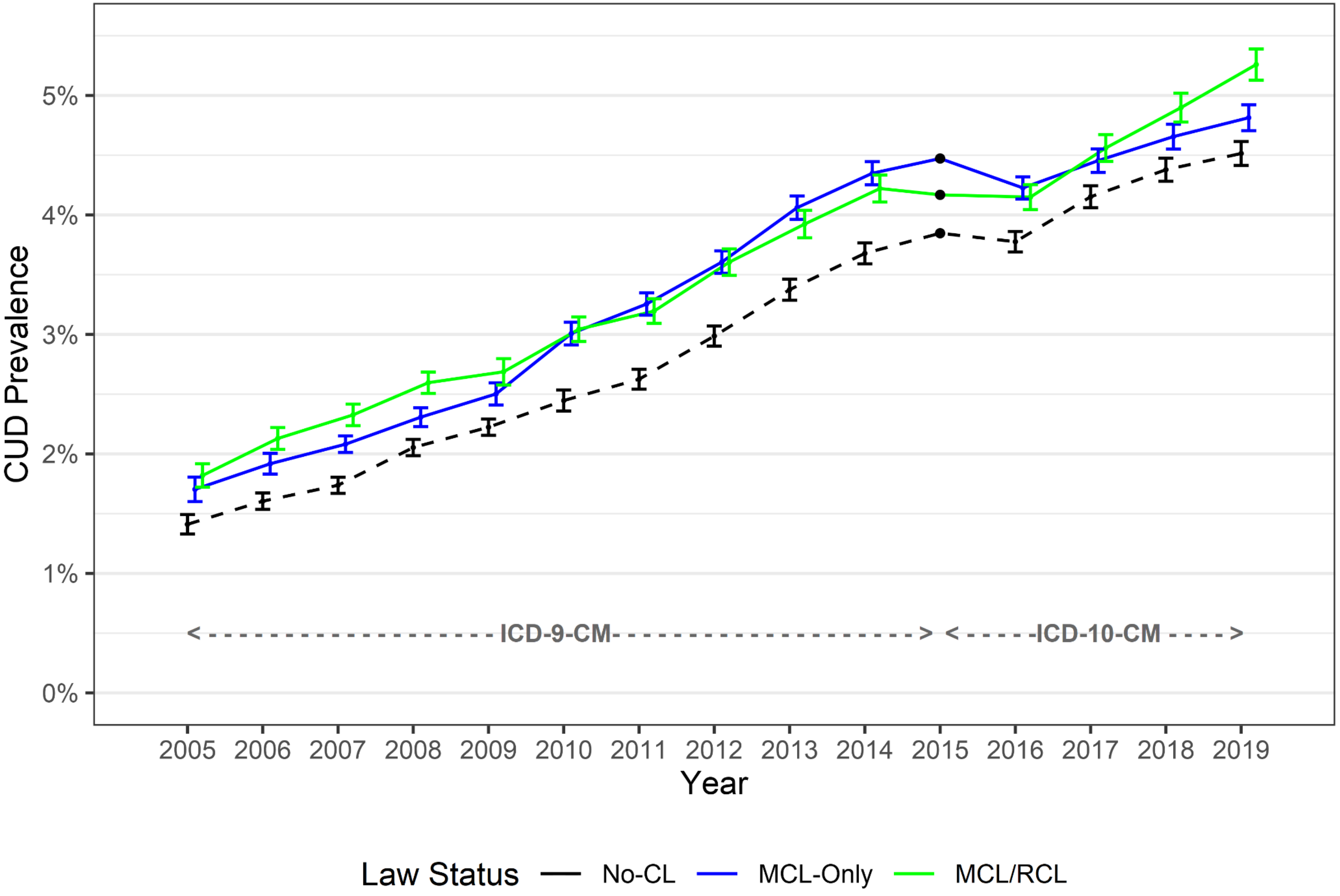

[a] Weighted mean prevalence estimates adjusted for age, sex, race/ethnicity and time-varying state covariates. For January 1, 2005, through September 30, 2015, ICD-9-CM diagnostic codes were used; for October 1, 2015, through December 31, 2019, ICD-10-CM codes were used. Note that estimates in 2015 represent a mix of diagnoses

© 2023 Hasin DS et al. *JAMA Psychiatry*.

collected using ICD-9-CM and ICD-10-CM due to the system-wide transition on October 1, 2015. Error bars indicate 95% confidence intervals; confidence intervals are very small because of the large sample sizes. VHA=Veterans Health Administration.

eFigure 1b. Trends in prevalence of CUD diagnoses from 2005 to 2019 (adjusted weighted mean estimates[a]), aggregated within the three groups of states defined by their cannabis legalization status at the end of 2019: Patients age 35-64

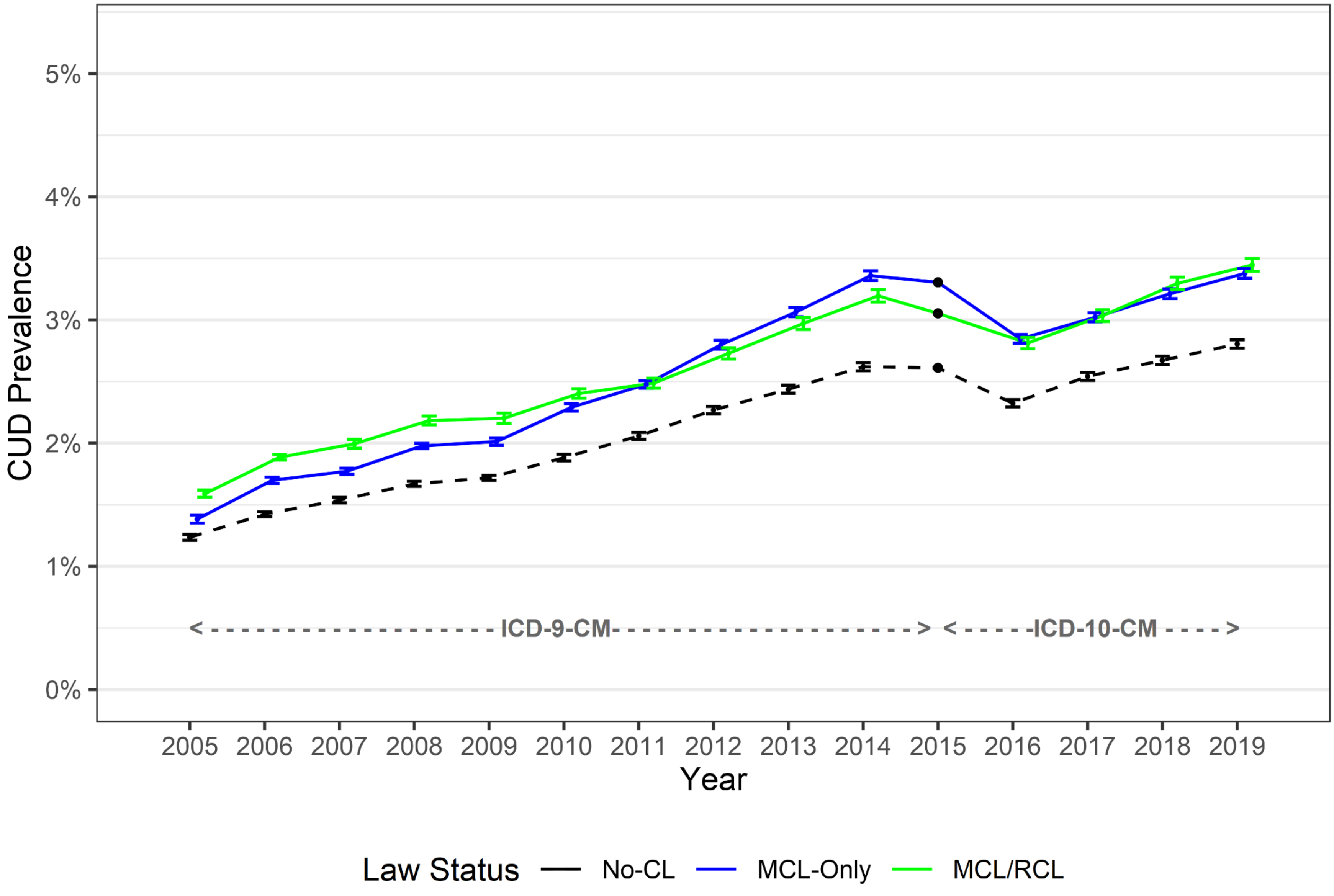

[a] Weighted mean prevalence estimates adjusted for age, sex, race/ethnicity and time-varying state covariates. For January 1, 2005, through September 30, 2015, ICD-9-CM diagnostic codes were used; for October 1, 2015, through December 31, 2019, ICD-10-CM codes were used. Note that estimates in 2015 represent a mix of diagnoses

© 2023 Hasin DS et al. *JAMA Psychiatry*.

collected using ICD-9-CM and ICD-10-CM due to the system-wide transition on October 1, 2015. Error bars indicate 95% confidence intervals; confidence intervals are very small because of the large sample sizes. VHA=Veterans Health Administration.

© 2023 Hasin DS et al. *JAMA Psychiatry*.

eFigure 1c. Trends in prevalence of CUD diagnoses from 2005 to 2019 (adjusted weighted mean estimates[a]), aggregated within the three groups of states defined by their cannabis legalization status at the end of 2019: Patients age 65-75

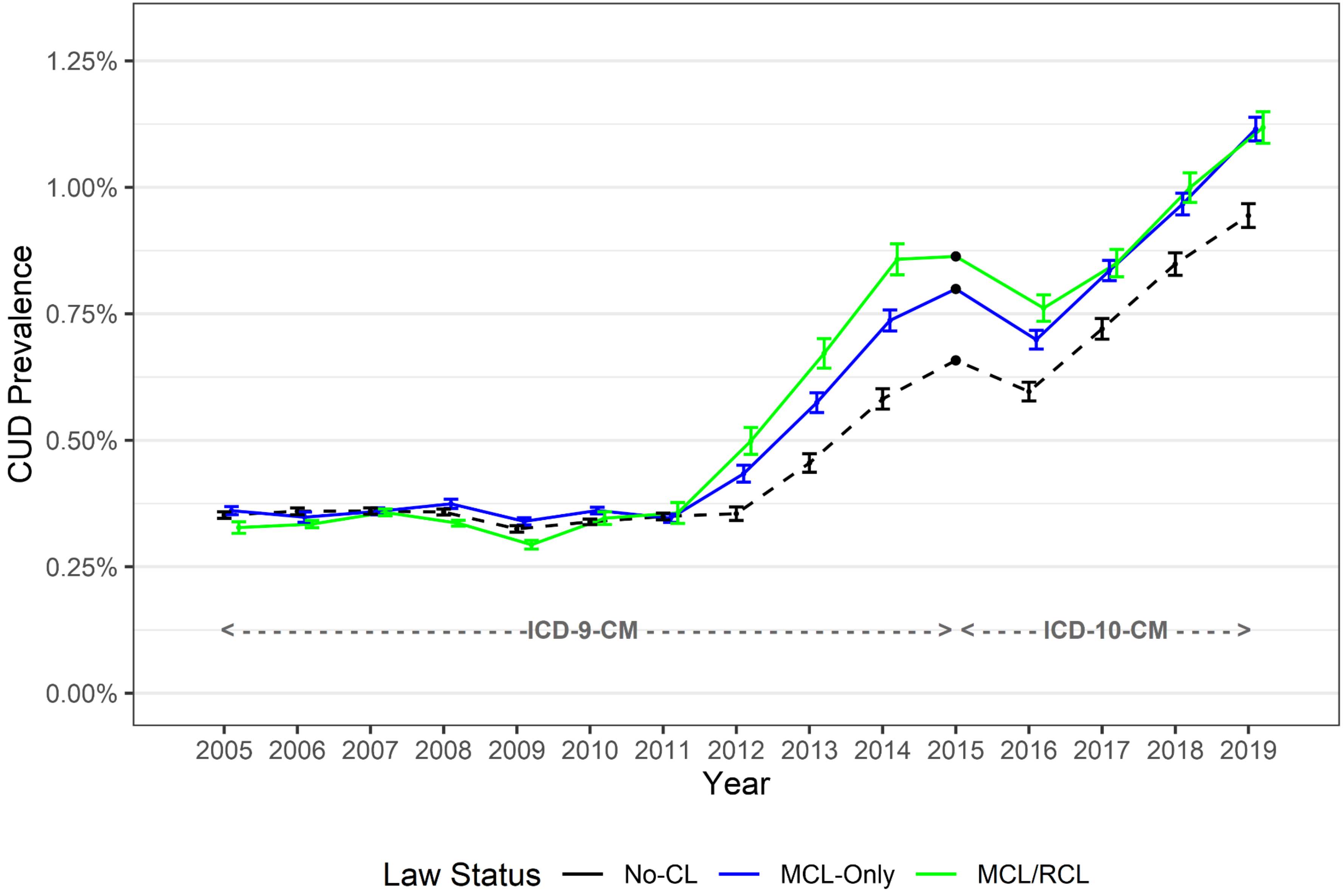

[a] Weighted mean prevalence estimates adjusted for age, sex, race/ethnicity and time-varying state covariates. For January 1, 2005, through September 30, 2015, ICD-9-CM diagnostic codes were used; for October 1, 2015, through December 31, 2019, ICD-10-CM codes were used. Note that estimates in 2015 represent a mix of diagnoses

© 2023 Hasin DS et al. *JAMA Psychiatry*.

collected using ICD-9-CM and ICD-10-CM due to the system-wide transition on October 1, 2015. Error bars indicate 95% confidence intervals; confidence intervals are very small because of the large sample sizes. VHA=Veterans Health Administration.

© 2023 Hasin DS et al. *JAMA Psychiatry*.

eFigure 2. State-specific effects of MCL and RCL enactment on CUD prevalence in VHA patients stratified by age

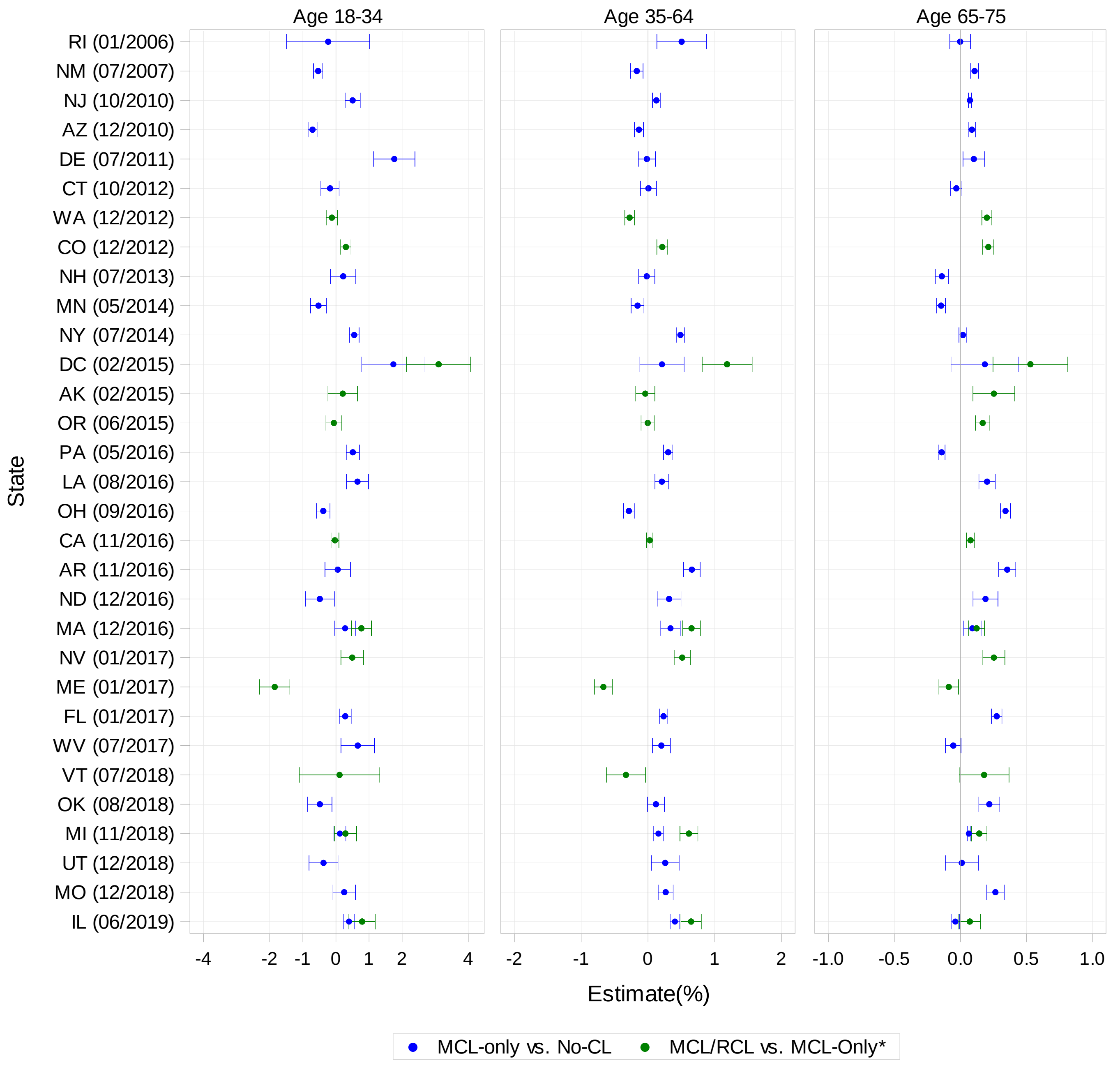

* In states that changed to MCL and RCL during the period, the MCL/RCL effect plotted is compared to No-CL for comparison with the MCL vs No-CL effect.

[a]Shown in the figure: point estimates and 95% confidence intervals from the Staggered-Adoption Difference-in-Difference (DID) regression models. Estimated effects represent absolute increase (positive values) or decrease (negative values) in CUD prevalence due to law enactment. Confidence intervals not including 0.0 indicate significant changes. Note that three states and DC changed from no-CL to MCL-only and then later MCL-only to MCL/RCL between 2005-2019. Their dates of MCL enactment: MI, 12/2008; DC, 07/2010; MA, 01/2013; and IL, 01/2014. The MCL/RCL effects shown for MI, DC, MA and IL in Figure 2 is compared to no-CL to facilitate comparison with the other MCL-only effects shown. The estimate of the MCL/RCL vs MCL-only effect is obtained by comparing the green (MCL/RCL) to blue (MCL-only) effect in those states.

© 2023 Hasin DS et al. *JAMA Psychiatry.*

eAppendix.

For epidemiological and large-scale healthcare system surveillance, distinguishing between true change in prevalence and change related to diagnostic algorithms used to identify the condition of interest in the study data source is important. This includes the impact of transitory disruptions, for example, the transition from ICD-9-CM and ICD-10-CM, which officially occurred on October 1, 2015[2]. This change and some disruption due to it was anticipated long in advance by the VHA[3], with active preparations ongoing during the four years prior to the transition[3].

In the general population, the prevalence of CUD did not decrease in 2015-2016[4,5], but these studies were based on DSM-IV criteria[6], not ICD-9-CM or ICD-10-CM criteria. However, such a decrease in CUD did occur during 2015-2016 in VHA patients, as we reported previously[2]. This decrease was also found in the present study in the no-CL, MCL-only and RCL states (Figure 1). No plausible substantive explanation exists for the 2015-2016 decrease in VA CUD rates. However, this decrease did coincide with the VA-wide transition from ICD-9-CM to ICD-10-CM. This transition generally presented many methodological challenges for EHR-based health research[7].

Studies of the effects of the ICD-9-CM to ICD-10-CM transition on rates of medical conditions have been inconsistent. In a study of 32 medical conditions in administrative claims data from the Taiwan National Health Insurance Research Database (NHIRD), about 2/3 of the conditions exhibited significant changes in both level and trend across the transition from ICD-9-CM to ICD-10-CM[8]. In a study of a 20% random sample of VA patients who used any VA inpatient or outpatient facility from 2014-2016, codes were created corresponding to ICD-9-CM and ICD-10-CM, and consistency in rates of 34 common chronic conditions during 2014-2016 were compared. Although most conditions did not show marked perturbances in rates across the years examined, the exceptions to this included alcohol dependence, drug dependence and tobacco/nicotine dependence[9], for which the authors did not offer an explanation.

Other studies specifically focused on effects of the ICD-9-CM to ICD-10-CM transition for alcohol[10], opioid[11], and cannabis use disorders[12] were conducted in acute care patients, i.e., those who were hospitalized[9-12] or treated in emergency departments. These studies showed increases rather than decreases in prevalence after the ICD-10-CM transition, attributed to provider use of the greater number of relevant categories available in ICD-10-CM. However, the clinical settings in which diagnoses were made for these hospitalized or emergency department patients differ considerably from the clinical situations of most VHA patients, who are seen on an ongoing outpatient basis in a large integrated health care system.

Seeking to understand the decrease after the transition to ICD-10-CM, we considered provider behaviors. VHA medical providers must add ICD codes to the EHR for new conditions and such codes do not disappear, e.g., for resolved or remitted conditions, unless they are actively removed. For cannabis use disorder, the definition of remission may be unclear and its occurrence challenging for some providers to assess. Thus, during the years that ICD-9-CM was used, active removal of cannabis use disorder diagnostic codes may not have seemed warranted, leaving a diagnosis entered at an earlier time that was automatically carried forward from year to year. However, after the ICD-10-CM transition, all ICD-9-CM codes were removed, and providers were required to reenter diagnostic codes for current conditions into the EHR. At that point in time, if current cannabis use disorder symptoms were not obvious, no code for ICD-10-CM cannabis use disorder would have been entered, resulting in an apparent drop in cannabis use disorder diagnoses. Subsequently, after the VHA completed the transition to ICD-10-CM in 2016, new or clearly continuing cannabis use disorder cases would be entered by providers. As our findings have clearly demonstrates, the 4-year ICD-10-CM period (2016-2019) was characterized by steady increases in cannabis use disorder diagnoses across age, sex, and racial/ethnic groups[5], and by state cannabis legal status (Figure 1), indicating that once the ICD-10-CM transition was completed, cannabis use disorder diagnoses in VHA patients continued to increase. Thus, the decrease in CUD rates in 2015-2016 appears best explained by a methodological factor, the transition from ICD-9-CM to ICD-10-CM.

eReferences

1. Athey S, Imbens GW. Design-based analysis in Difference-In-Differences settings with staggered adoption. *Journal of Econometrics.* 2022;226(1):62-79.
2. Center for Disease Control and Prevention. International Classification of Diseases, (ICD-10-CM/PCS) Transition - Background. https://www.cdc.gov/nchs/icd/icd10cm_pcs_background.htm. Published 2015. Accessed Nov 23, 2022.
3. Weems S, Heller P, Fenton SH. Results from the Veterans Health Administration ICD-10-CM/PCS Coding Pilot Study. *Perspect Health Inf Manag.* 2015;12(Summer):1b.
4. Center for Behavioral Health Statistics and Quality. Results from the 2016 National Survey on Drug Use and Health: Detailed Tables. https://www.samhsa.gov/data/sites/default/files/NSDUH-DetTabs-2016/NSDUH-DetTabs-2016.pdf. Published 2017. Accessed Nov 23, 2022.
5. Hasin DS, Saxon AJ, Malte C, et al. Trends in Cannabis Use Disorder Diagnoses in the U.S. Veterans Health Administration, 2005-2019. *Am J Psychiatry.* 2022:appiajp22010034.
6. American Psychiatric Association. *Diagnostic and statistical manual of mental disorders (4th ed., Text Revision).* Washington, DC2000.
7. Singh JA. Time-trends in hospitalizations with cannabis use disorder: A 17-year U.S. national study. *Subst Abus.* 2022;43(1):408-414.
8. Hsu MC, Wang CC, Huang LY, Lin CY, Lin FJ, Toh S. Effect of ICD-9-CM to ICD-10-CM coding system transition on identification of common conditions: An interrupted time series analysis. *Pharmacoepidemiol Drug Saf.* 2021;30(12):1653-1674.
9. Yoon J, Chow A. Comparing chronic condition rates using ICD-9 and ICD-10 in VA patients FY2014-2016. *BMC Health Serv Res.* 2017;17(1):572.
10. Heslin KC, Barrett ML. Shifts in Alcohol-Related Diagnoses After the Introduction of International Classification of Diseases, Tenth Revision, Clinical Modification Coding in U.S. Hospitals: Implications for Epidemiologic Research. *Alcohol Clin Exp Res.* 2018;42(11):2205-2213.
11. Heslin KC, Owens PL, Karaca Z, Barrett ML, Moore BJ, Elixhauser A. Trends in Opioid-related Inpatient Stays Shifted After the US Transitioned to ICD-10-CM Diagnosis Coding in 2015. *Med Care.* 2017;55(11):918-923.
12. Hall KE, Yang H, Goulding D, Contreras E, James KA. Interrupted time series analysis of cannabis coding in Colorado during the ICD-10-CM transition. *Inj Prev.* 2021;27(S1):i66-i70.

© 2023 Hasin DS et al. *JAMA Psychiatry.*
