# Supplementary material for: State Cannabis Legalization and Cannabis Use Disorder in the US Veterans Health Administration, 2005 to 2019
Source: JAMA Psychiatry. 2023 Mar 1;80(4):380–8. doi: 10.1001/jamapsychiatry.2023.0019 (PMC9979011; doi:10.1001/jamapsychiatry.2023.0019)
Supplement: Supplement 2. — Data sharing statement [file jamapsychiatry-e230019-s002.pdf]

Data Sharing Statement

Hasin. State Cannabis Legalization and Cannabis Use Disorder in the US Veterans Health Administration, 2005 to 2019. *JAMA Psychiatry*. Published March 01, 2023. doi:10.1001/jamapsychiatry.2023.0019

Data
Data available: No
